# The association between silica exposure, silicosis and tuberculosis: a systematic review and meta-analysis

**DOI:** 10.1186/s12889-021-10711-1

**Published:** 2021-05-20

**Authors:** Rodney Ehrlich, Paula Akugizibwe, Nandi Siegfried, David Rees

**Affiliations:** 1 School of Public Health and Family Medicine, University of Cape Town, Cape Town, South Africa; 2 Independent Clinical Epidemiologist, Cape Town, South Africa; 3 Department of Psychiatry and Mental Health, University of Cape Town, Cape Town, South Africa; 4 National Institute for Occupational Health, Johannesburg, South Africa; 5 School of Public Health, University of the Witwatersrand, Johannesburg, South Africa

## Abstract

**Background:**

While the association between occupational inhalation of silica dust and pulmonary tuberculosis has been known for over a century, there has never been a published systematic review, particularly of experience in the current era of less severe silicosis and treatable tuberculosis. We undertook a systematic review of the evidence for the association between (1) silicosis and pulmonary tuberculosis, and (2) silica exposure and pulmonary tuberculosis controlling for silicosis, and their respective exposure-response gradients.

**Methods:**

We searched PUBMED and EMBASE, and selected studies according to a priori inclusion criteria. We extracted, summarised and pooled the results of published case-control and cohort studies of silica exposure and/or silicosis and incident active tuberculosis. Study quality was assessed on the Newcastle-Ottawa Scale. Where meta-analysis was possible, effect estimates were pooled using inverse-variance weighted random-effects models. Otherwise narrative and graphic synthesis was undertaken. Confidence regarding overall effect estimates was assessed using the GRADE schema.

**Results:**

Nine studies met the inclusion criteria. Meta-analysis of eight studies of silicosis and tuberculosis yielded a pooled relative risk of 4.01 (95% confidence interval (CI) 2.88, 5.58). Exposure-response gradients were strong with a low silicosis severity threshold for increased risk. Our GRADE assessment was high confidence in a strong association. Meta-analysis of five studies of silica exposure controlling for or excluding silicosis yielded a pooled relative risk of 1.92 (95% CI 1.36, 2.73). Exposure-response gradients were observable in individual studies but not finely stratified enough to infer an exposure threshold. Our GRADE assessment was low confidence in the estimated effect owing to inconsistency and use of proxies for silica exposure.

**Conclusions:**

The evidence is robust for a strongly elevated risk of tuberculosis with radiological silicosis, with a low disease severity threshold. The effect estimate is more uncertain for silica exposure without radiological silicosis. Research is needed, particularly cohort studies measuring silica exposure in different settings, to characterise the effect more accurately as well as the silica exposure threshold that could be used to prevent excess tuberculosis risk.

**Supplementary Information:**

The online version contains supplementary material available at 10.1186/s12889-021-10711-1.

## Background

Occupational silica exposure continues to occur in almost all countries and in many industries and occupations: construction, agriculture and mining being among the largest employers. Consequently, millions of individuals are at risk of silica-associated disease. In South Africa between 1973 and 2013 an estimated minimum 1.2 million workers passed through the gold mining industry with its high silica dust exposures [1, 2]. Of other middle income countries India has approximately 11.5 million people working in silica-exposed jobs, Brazil over 2 million, while China is thought to have the largest number of silicosis cases, with 6000 new cases reported annually [3].

Tuberculosis is an infectious disease caused by *Mycobacterium tuberculosis*, most commonly affecting the lung. It may also occur in the form of latent tuberculosis, a dormant state which may progress to active disease many years after the initial infection. All of the countries mentioned above are classified by the World Health Organization (WHO) as high burden tuberculosis countries [4].

While the association between certain dusty occupations and “consumption” or “phthisis” was recognised in the nineteenth Century (C.), the acceptance of silicosis, the fibrotic lung disease due to silica dust inhalation, and pulmonary tuberculosis as distinct diseases dates from the early twentieth C. [5–8].Their aetiological relationship became widely accepted in settings where high silica exposures and untreatable tuberculosis prevailed [8–10]. However, doubts were still raised at public forums [11], including by the chief medical spokesperson of the South African gold mining industry between the 1930s and 1950s [12]. Whatever the case, with the decline in silicosis occurrence in high-income countries by the second half of the twentieth century owing to more effective dust control [13–15], the decline in tuberculosis transmission in such countries [16] and the advent of antituberculous chemotherapy, interest in the association and its implications waned.

However, silica exposure, silicosis and tuberculosis continue to co-occur in many working populations worldwide. There has recently been a renewal of international interest in the association as part of global efforts to stem the tuberculosis epidemic [17, 18]. The huge toll of silicosis and tuberculosis on South African gold miners over the past few decades has been extensively described [19, 20]. Co-occurrence of silicosis and tuberculosis is frequently reported from other high tuberculosis-burden countries with both small scale mineral working and large scale extractive industries – in India [21, 22], China [23] and Russia [24]. The association also remains relevant to low tuberculosis burden countries such as Portugal [25].

Despite the subject's continuing importance, reviews from the second half of the twentieth C. onwards which focus on the association between silica, silicosis and tuberculosis are scarce (e.g. [26]). There has never to our knowledge been a systematic review. More general reviews of silica and disease [8, 27–30] vary in their treatment of the silica exposure/silicosis-tuberculosis association, usually with limited critical attention to some important considerations. These include the distinction between silicosis and silica exposure in the absence of silicosis, the implication of a substantial proportion of silicosis being undetectable on the chest radiograph [31, 32] and the differences between studies of tuberculosis incidence and tuberculosis mortality as the outcome. Focus is typically on tuberculosis as a clinical complication of silicosis rather than on the shape and size of exposure-relationships between silica and tuberculosis that would enable thresholds to be discerned for prevention purposes. Examination of the quality of primary studies is generally absent.

Unanswered questions therefore remain with important implications for policies and practices to protect silica-exposed workers. In particular, are current standards for control of workplace silica exposure sufficient to prevent increased tuberculosis risk irrespective of whether silicosis is detectable on the chest x-ray? This requires knowledge of the shape of the silica-tuberculosis exposure-response gradient and the threshold above which excess tuberculosis risk attributable to dust would be controlled.

The association is relevant also to workers’ compensation for tuberculosis in industries with a silica hazard. The International Labour Organization (ILO) List of Occupational Diseases [33] includes silicotuberculosis (silicosis complicated by tuberculosis) and tuberculosis alone without specification of causal exposures. It is therefore unclear whether tuberculosis in silica-exposed workers in the absence of radiological silicosis is included. A review of compensation practices regarding miners’ occupational lung disease in a number of countries suggests that South Africa is one of the few or perhaps the only country where tuberculosis is recognised as an occupational disease in miners in the absence of radiological silicosis [34].

In this inquiry, we aimed to systematically review the evidence for the association between (1) silicosis and pulmonary tuberculosis, and (2) silica exposure and pulmonary tuberculosis excluding or controlling for radiological silicosis. Exposure-response gradients were examined for both objectives.

## Methods

### Systematic review and selection criteria

We carried out a systematic review to assess the evidence from human controlled studies conducted from the 1970s onwards for the associations between silica, silicosis and tuberculosis in adults. We excluded laboratory studies. We registered the protocol with PROSPERO (registration identification CRD 4201912696). Protocol development and review reporting were guided by the Preferred Reporting Items for Systematic Reviews and Meta-Analyses (PRISMA) checklist [35].

Inclusion criteria, grouped according to the population–exposure–comparator-outcome (PECO) framework [36], were as follows:

- Population - populations in which individuals were or had been exposed to crystalline silica dust and in which incident tuberculosis was recorded;
- Exposures - studies of individuals with silicosis; and/or with respiratory exposure to silica dust, explicitly measured or inferred from occupation or industry;
- Comparators - studies reporting comparative effect estimates, specifically case-control or cohort studies reporting risk, rate or odds across groups exposed to different levels of silica (including binary comparisons of exposed/unexposed), and across groups with silicosis (including different grades) and without;
- Outcome - studies reporting incident active pulmonary tuberculosis, with or without extrapulmonary tuberculosis. Diagnosis must have been made on histological or microbiological grounds, or an explicit combination of clinical assessment, radiology and/or response to treatment. Tuberculosis must have been diagnosed after the onset of silica exposure or silicosis diagnosis;
- English-language full-text available;
- Publication 1970 to April 2020 inclusive.

We excluded studies without comparison between higher and lower (or zero) levels of silica exposure or with insufficient information to assign or impute different levels of silica exposure; and for silicosis, without a comparison between those with and without the disease. We also excluded studies of latent tuberculosis infection or of tuberculosis self-reported or based on radiology alone, cross-sectional studies and mortality studies, solely autopsy based studies, and studies of tuberculosis cases from registers without the diagnostic method specified.

### Search terms, sources and strategy

We searched the following healthcare and biomedical electronic databases up to 30 April 2020 using a comprehensive search strategy as outlined in Table S1: (1) PUBMED via http://www.ncbi.nlm.nih.gov/pubmed; and (2) EMBASE via www.embase.com. The strategy was translated into the appropriate syntax for each database (Additional File 1, Table S1). The search strategy included database-specific and free text terms for [silicosis] and [tuberculosis] and was not limited by study design filters, language, or publication date. We checked reference lists of studies screened as relevant, as well as review articles for additional relevant citations. Where necessary we contacted authors of published studies for information.

### Study selection

**Fig. 1:**
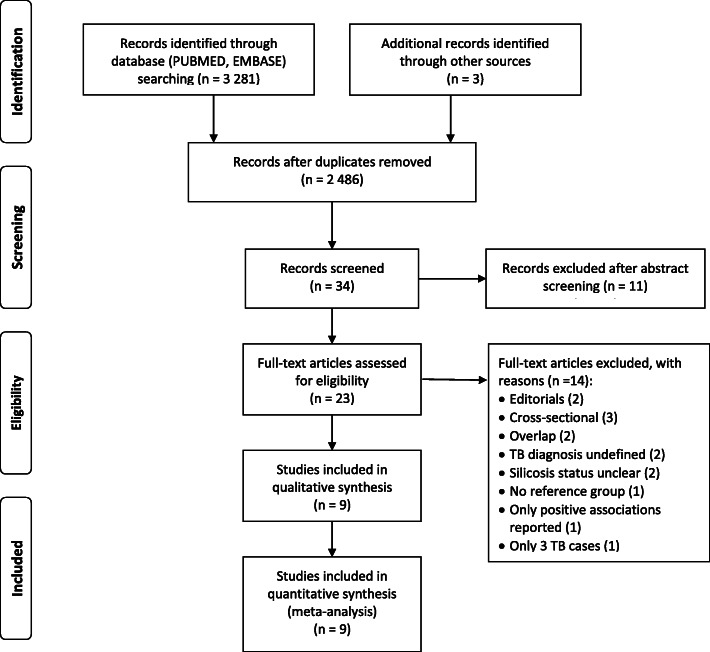
Flow diagram of search and selection

The selection process is presented in Fig. 1. Duplicate records were identified and removed. Two authors with expertise in the subject (RE, DR) independently identified potentially eligible studies based on article abstracts, applying the inclusion and exclusion criteria above. Full-text articles were obtained for further independent eligibility assessment. Disagreement was settled through joint re-assessment of the article and discussion with a third reviewer (NS).

### Data extraction

Data from alternate articles were extracted by two of the authors (DR, RE) using a piloted template, checked by the other and disagreement resolved by discussion with NS. Data included first author, publication date, location/industry, study design, study population, calendar period of study, number of individuals included [i.e. silica exposed and unexposed, with and without silicosis (including grades of exposure/disease where available), and with and without tuberculosis], method of silica exposure estimation and/or silicosis identification, method of tuberculosis diagnosis, confounders measured and/or controlled for, and overall measure of effect (relative risk - rate, risk or hazard ratio, or odds ratio) by exposure or diagnosis category, both unadjusted and adjusted where available, with 95% confidence intervals. Where degree of silica exposure or silicosis was stratified, stratum-specific measures of association were extracted.

### Study quality assessment

Risk of bias of each eligible study was assessed independently by two authors (RE and DR) using the Newcastle Ottawa Scale (NOS) for case-control and cohort studies, and disagreements settled through discussion and consultation with a third author (NS). The NOS was developed by the University of Newcastle, Australia, and the University of Ottawa, Canada, to assess the quality of nonrandomized studies included in systematic reviews [37]. There was no modification of the instrument for this review. The questions and score assignment for each study design are included as Table S2 (Additional File 1). Quality assessment scores were not used to exclude studies.

Confounding by risk factors for tuberculosis likely to be associated independently with silica exposure or silicosis, was considered in relation to study design. In these occupational (adult) cohorts, age, and in some settings HIV, were judged to be the most important potential confounders [38, 39]. Additional confounders of relevance, particularly in cohort studies that were population rather than industry based, or where the general population was used as the reference population, included smoking, congregate settings (in transport, housing and the workplace), socioeconomic status, undernutrition and indoor air pollution [40–42]. Heavy alcohol intake and diabetes were considered as potential confounders as they are established risk factors for tuberculosis [41, 42]. Finally, bias arising from an increased likelihood of investigating for and diagnosing tuberculosis among those with silica exposure or silicosis was considered as a potential selection bias.

### Standardisation of results and summary presentation

Where studies were sufficiently clinically and methodologically homogeneous, we conducted a meta-analysis for the selected outcome of tuberculosis. Aggregated participant data were used for data synthesis. Meta-analysis was conducted on RevMan version 5.3 [43] using the random effects model given anticipated statistical heterogeneity. We assessed the presence of heterogeneity in study results using the chi-square test and quantified the degree of heterogeneity using the I^2^ statistic.

We preferentially report on the adjusted analysis using the estimate of effect reported in the study rather than calculating estimates of effects based on the crude data. Where only crude data were presented, we calculated the crude risk ratio and 95% confidence interval for dichotomous data if appropriate, and combined these with the adjusted estimates using the generic inverse variance function in RevMan 5.3 [43]. We explored anticipated heterogeneity by tuberculosis burden in country of study, i.e. high burden versus low and intermediate burden as defined by the WHO [4]. We further examined heterogeneity by study design (cohort vs case-control) and conducted a sensitivity analysis by examining the impact on overall findings of removing studies at risk of bias.

For those studies where we were not able to pool data, we provide a narrative and graphic synthesis using histograms showing relative risk or odds over different metrics for the individual studies. Disease severity-response gradients were analysed by extent of silicosis, and silica exposure-response relationships using stratified exposure metrics (e.g. cumulative dust exposure, duration of employment, or occupational dustiness category). To study the effect of silica exposure in the absence of silicosis, we included only studies which controlled for silicosis in the analysis though adjustment or excluded those with the diagnosis.

### Evidence synthesis and assessment of certainty

GRADE was used to judge the overall quality of the evidence with data directly imported from Revman into GRADEPro (GRADEpro Guideline Development Tool. McMaster University, 2020) [44]. Using this instrument in our context, overall quality reflects our confidence that the effect estimates are adequate to support an aetiological inference. Owing to the observational nature of the included studies, the overall confidence commences as low on the GRADE schema. We considered the following characteristics to mark quality up or down: risk of bias (individual study limitations); consistency, directness and precision of the evidence; and publication bias and selective reporting. We also considered the following as reasons to mark upwards: the magnitude of the effect, exposure-response gradient, and the likelihood that any residual confounding would have reduced rather than exaggerated the true effect [45].

## Results

### Study selection

The PUBMED and EMBASE searches yielded 1674 and 1607 records respectively. Following electronic deduplication, one author (NS) reviewed all potentially duplicate records and removed 798 true duplicates, resulting in a total of 2483 records (Fig. 1). From these, two of the authors (RE, DR) identified 34 potentially eligible records, of which 23 full text articles were obtained for full eligibility assessment. Fourteen of the 23 articles were excluded after assessment (Additional File 1, Table S3). Nine articles reporting on separate studies met the inclusion criteria.

### Study characteristics

Table 1 sets out the characteristics of the nine included studies [46–54]. There were two case-control studies [50, 54], and seven cohort studies [46–49, 51–53]. Publication ranged from 1986 to 2013 and included five low or intermediate tuberculosis burden countries, Sweden, Denmark, Taiwan, Hong Kong (prior to unification with China), and Iran; and only one high tuberculosis country, South Africa, predominating with four studies. There were six industry-specific studies, including mining and quarrying (46, 48–51] and foundries (46, 47]; one regional population study [54]; one based on a national kidney disease register [53]; and one on a national silicosis register [52].

**Table 1:** Silica exposure, silicosis and tuberculosis: study characteristics, sample size, and definition of exposure and outcome

First author, publication year. Country^a^, study dates	Study design (effect measure), study population and data sources for comparison groups	Sample size	Tuberculosis (TB) diagnosis	Silica exposure/silicosis ^b^ categories compared, diagnostic/exposure assessment
Westerholm 1986 [46]. Sweden, 1959–1977.	*• Matched retrospective cohort (risk ratio)* *•* Male silicosis cases reported 1959–1977 to the National Swedish Pneumoconiosis Register from mining, quarrying and tunneling industries, and iron and steel foundries. Controls from silica exposed persons undergoing periodic health examinations recorded in same register. *•* TB identified from the Swedish Tuberculosis Index 1971–1980.	712 silicosis, 810 no silicosis, matched for occupation, age and calendar year at first silica exposure.	Verified by microscopy or by guinea pig or bacteriological culture.	*•* Silicosis versus no silicosis*.* *•* Diagnostic criteria for silicosis not reported.
Sherson 1990 [47]. Denmark, 1967–1986.	*• Retrospective cohort (standardized incidence ratio)* *•* Male foundry workers in the Foundry Worker Registry of the Danish Labour Inspectorate *•* Registry populated with data from two national silicosis surveys, 1967–1969 and 1972–1974. *•* Pulmonary TB identified from the Danish TB Registry though 1986.	155 silicosis, 5424 no silicosis.	19/21 cases had positive cultures. Diagnostic criteria not stated for the other two.	*•* (1) Silicosis versus no silicosis. (2) Years of metal foundry work. *•* Diagnostic criteria for silicosis not reported.
Cowie 1994 [48]. South Africa, 1984–1991.	*• Retrospective cohort (annual incidence rate; risk ratio)* *•* Medical surveillance database of 90,000 black male gold miners from 24 mines in the Orange Free State Province. *•* Silicosis and controls ascertained prospectively. *•* TB identified from a central TB registry.	818 silicosis, 335 no silicosis, matched for day of CXR and age.	Positive sputum cultures in all pulmonary TB subjects. 30 cases of extrapulmonary TB (24 intrathoracic).	*•* (1) Silicosis vs no silicosis; (2) increasing ILO profusion of radiologic silicosis: 0, 1, 2, 3. *•* Silicosis read on full size CXR using ILO Classification.
Hnizdo 1998 [49]. South Africa, 1968/71–1995.	*• Prospective cohort (rate ratio, relative risk)* ^c^ *•* White male gold miners aged 45–54 years with ≥10 yr underground experience who attended state examination bureau for compulsory medical surveillance 1968–1971. *•* Silicosis identified from ongoing surveillance CXRs and/or at autopsy in decedents. TB identified from medical and/or autopsy records through 1995.	2255 miners; 321 radiological silicosis, 719 autopsy silicosis (of whom 546 autopsy silicosis only).	Positive sputum test (76), positive histology (36), and positive CXR (5).	*•* Silicosis vs no silicosis; (2) Quartiles of cumulative dust exposure (mg-yr/m^3^); (3) Degree of silicosis at autopsy. *•* Radiological silicosis ILO > 1/1, 1990 - re-reading of all previous CXRs. *•* Autopsy silicosis on microscopy by pathologist: none, negligible, slight, moderate/marked.
Corbett 1999 [50]. South Africa, 1993–1996.	*• Case-control (odds ratio)* *•* Silicosis cases and controls (trauma, surgical) from medical and personnel records of gold miners attending a company hospital, the sole source of tertiary care for this population. *•* TB cases: random sample of central database.	381 TB cases; 180 non-TB controls.	First episode of culture positive TB.	*•* (1) Silicosis grade ^d^ (none, “possible” = ILO 0/1, “probable =1/0, “early” =1/1, “high grade” = 1/1); (2) years of gold mining: < 10; 10–14; 15–19: ≥ 20; (3) dusty job at diagnosis. *•* Silicosis consensus by 2 readers on mini-CXRs in 93.4% of subjects, standard size films in the rest.
Corbett 2000 [51]. South Africa, 1991–1996.	*• Retrospective cohort (rate ratio)* *•* Medical and personnel records of male gold miners attending peripheral clinics and company hospital of a single gold mining company. *•* TB cases identified from a centralized TB database. *•* No overlap with Corbett 1999.	4022 miners 1025 silicosis (including ILO 0/1).	Pulmonary TB (80.8% of TB cases): smear or culture positive (88.4%) or compatible radiologic changes plus clinical and laboratory features consistent with TB.^e^	*•* Silicosis grade (none, ILO 0/1, 1/0, 1/1, > 1/1); (2) years of employment 0–4; 5–9; 10–19; ≥ 20; (3) main job underground vs surface. *•* Silicosis consensus read by 2 readers on mini-CXRs.
Chang 2001 [52]. Hong Kong, 1988–1999.	*• Retrospective cohort (relative risk; standardized risk ratio)* *•* Silicosis diagnosed at Pneumoconiosis Medical Board 1988–1993, followed for TB through records until death or end of 1999. *•* Supplementary information from TB Notifications Register.	707 silicosis	Bacteriologically confirmed cases.	*•* (1) Cohort vs general population; (2) within cohort: (a) years of occupational dust exposure; (b) Caisson construction work; ^f^ (c) ILO profusion > grade 1; (e) progressive massive fibrosis (PMF). *•* Diagnostic criteria for silicosis not stated.
Li 2011 [53]. Taiwan, 1998–2006.	*• Retrospective cohort (hazard ratio)* *•* Incident end stage renal disease (ESRD) patients recorded in National Health Insurance Research (NIHR) Database 1998–2004. *•* Incident TB identified from the Taiwan Centers for Disease Control TB database through 2006. *•* Silicosis recorded from co-morbidity information on NHIR database.	49,983 ESRD. 52 silicosis.	“ICD-9 code (010–018) in at least three ambulatory visits and insurance claims for anti-TB drugs … for more than 90 days.”	*•* Silicosis vs no silicosis. *•* Diagnostic criteria for silicosis not given.
Yarahmadi 2013 [54]. Iran, 2006–2011.	*• Case-control* ^g^ *(odds ratio)* *•* TB cases and controls (individuals investigated for TB and found to be negative) at the Infectious Disease Control Center of the Health Deputy of Khoramabad City, Lorestan.	871 TB cases (55.5% female); 429 non-TB controls (56.1% female).	Microscopy of 3 sputum samples of people coughing for 2 weeks or longer.	*•* Silica exposure (based on interview) without silicosis vs no silica exposure; *•* Silicosis: pulmonologist assessment of subjects with suggestive CXR, with HRCT if required.

*CXR* chest radiograph, *ESRD* end stage renal disease, *HRCT* high resolution computed tomography

^a^ Country TB burden classification: Low or intermediate: Denmark, Hong Kong, Iran, Sweden, Taiwan; High: South Africa

^b^ Radiological silicosis unless otherwise specified

^c^ Both terms used in study

^d^ ILO grade equivalents confirmed with author

^e^ Includes 29/213 smear and culture negative cases with diagnosis based on response to anituberculous treatment, radiology, compatible tissue or biochemical characteristics, etc.

^f^ “(A) cylindrical foundation which after excavation is concreted in place for the purpose of transferring structural load to the bedrock construction” [55]. Reported respirable dust range was 2.95–64.0 mg/m^3^ in absence of wet suppression and half that with wet suppression

^g^ Designated by authors as cross-sectional, but TB cases entered into a register over 2006–2011 were interviewed and then compared to non-cases (controls)

### Risk of bias assessment

**Fig. 2:**
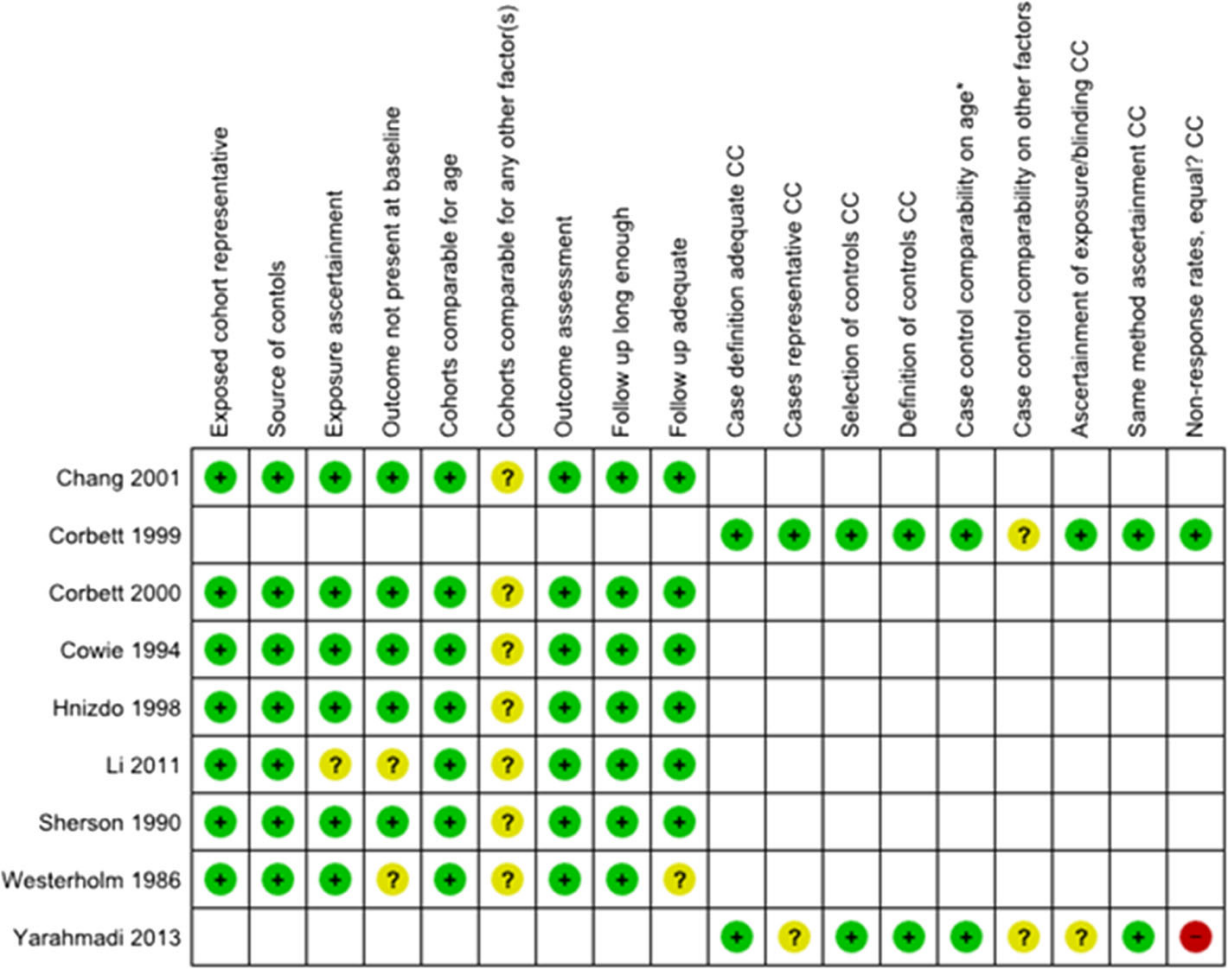
Risk of bias in nine studies of silica exposure, silicosis and tuberculosis

Figure 2 summarises the risk of bias in the reviewed studies by nine NOS characteristics, one set for case-control and one for cohort studies. In six of the studies [47–52], all criteria were assessed as low risk of bias except for comparability of the study groups on “any additional factor” (other than age, and interpreted here as controlling for relevant additional factors) which was assessed as a source of uncertain risk of bias. Based on a close analysis of confounding and selection bias (See Additional File 1: Note and Tables S4 and S5), we concluded that although studies dealt with confounding in different ways, the overall risk of bias in these six studies was plausibly low.

There were additional sources of uncertain bias in the remaining three studies. In the Swedish registry study [46], there was an 11-year gap between the inception of the silicosis cohort and the first ascertainment of tuberculosis, and no information on how tuberculosis was excluded at baseline. In the Taiwan study [53] based on an end stage renal disease register there was lack of information on definition of silicosis and on how tuberculosis at baseline was excluded. Finally, the Iranian community study [54] was judged as having a high risk of bias owing to low response rates, uncertain risk of bias in the representativeness of cases and the lack of blinding in interviewing cases and controls about past silica exposure.

Titles and abstracts reported in languages other than English were excluded from the review but were screened for relevance. A single study, in Czech, was identified on abstract (as the article was unobtainable) which might have qualified for inclusion [56].

### Studies of the association between silicosis and tuberculosis

**Fig. 3:**
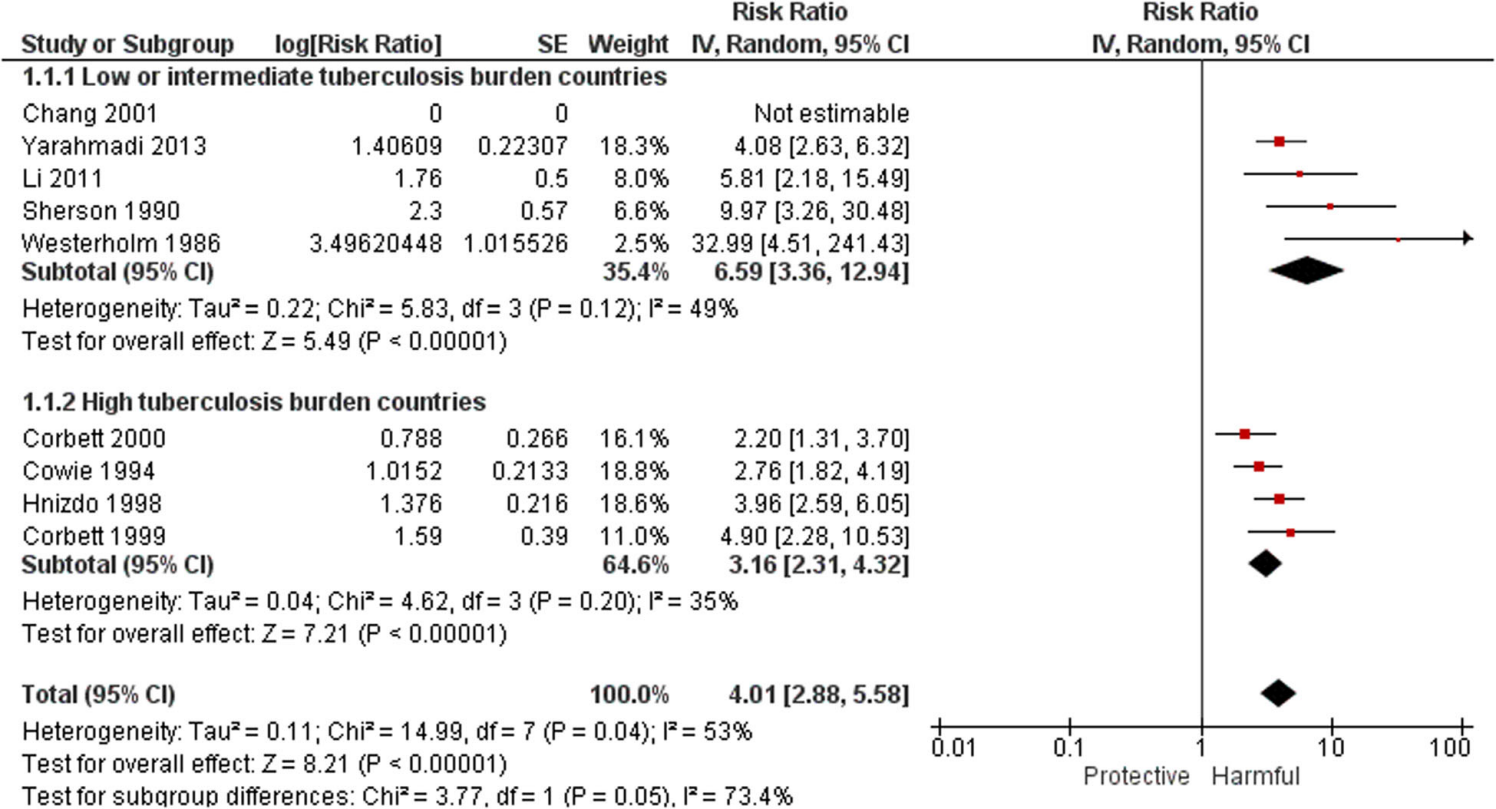
Forest plot: Studies of the association between silicosis and tuberculosis

Table 2 summarises the results of eight studies of silicosis as a binary exposure and Fig. 3 presents the meta-analysis. All except Yarahmadi et al. [54] adjusted for age but differed in their other covariates. All effect measures, both crude and adjusted, showed a substantial effect of silicosis, ranging from 2.2 [51] to 32.99 [46]. The summary relative risk, preferentially combining adjusted estimates where these were reported with crude estimates, was 4.01 (95% confidence interval (CI) 2.88, 5.58) with I^2^ = 53%, indicating moderate statistical heterogeneity. Given the large outlier effect size of Westerholm et al. [46], omission of this study resulted in a reduced summary relative risk of 3.70 (95% CI 2.78, 4.93) and statistical heterogeneity of 41%. Removing, in addition, the one study with an overall high risk of bias as described earlier [54] did not change the summary relative risk (3.69, 95% CI 2.59, 5.25).

**Table 2:** Tuberculosis risk or odds by silicosis relative to no silicosis or general population

First author, year of publication	Study Design	Study /control population(s)	N	Controlling for	Estimate (95% CI) (silicosis vs no silicosis)
**Low or intermediate TB burden countries**					
Westerholm 1986 [46]	Cohort	Mining, quarrying and tunneling industries and iron and steel foundries.	1522	Occupation, age, calendar year at first silica exposure.	OR 32.99 (4.50, 241.58) ^a^
Sherson 1990 [47]	Cohort	Foundry workers, general population	5579	(i)None (ii)Age	(i) RR 8.25 (2.81, 24.25) ^b^ (ii) SIR 10.00 (2.72, 25.61)
Chang 2001 [52]	Cohort	Silicosis register / general population	707	Age, gender	SIR 4.9 ^c^
Li 2011 [53]	Cohort	End-stage renal disease patients	49,983	Age, gender, income,COPD	HR 5.82 (2.17, 15.6)
Yarahmadi 2013 [54]	Case control	Community		None	OR 4.08 (2.63, 3.62)
**High TB burden countries**					
Cowie 1994 [48]	Cohort	Gold miners	1153	Age, date of CXR	RR 2.8 (1.9, 4.1)
Hnizdo 1998 [49]	Cohort	Gold miners	2255	Age, smoking, cumulative dust exposure	RR 4.18 (2.75, 6.36)
Corbett 1999 [50]	Case control	Gold miners	561	Age, HIV, duration, employed, dusty job	OR 4.90 (2.32,10.58)
Corbett 2000 [51]	Cohort	Gold miners	4022	Age, HIV, duration employed, surface/underground	RR 2.2 (1.3, 3.7) ^d^

*CI* confidence interval, *OR* odds ratio, *RR* relative risk, rate ratio or risk ratio (see Table 1); SIR, standardised incidence ratio; HR, hazard ratio;

CXR, chest x-ray

^a^ Estimated for this review

^b^ (i) RR and CI estimated for this review comparing silicotics with non-silicotics in the same cohort; (ii) indirect standardization using general population control, presented here with base 1 rather than 100

^c^ No CI provided

^d^ OR for silicosis > 1/1 not provided. OR for ILO 1/1 used as proxy

The summary relative risk for studies in low and intermediate burden tuberculosis countries was 6.59 (95% CI 3.36, 12.94) with I^2^ = 49%, indicating moderate statistical heterogeneity. Omission of Westerholm et al. [46] reduced this summary relative risk to 4.97 (95% CI 3.18, 7.77) and statistical heterogeneity to 14%. In high tuberculosis burden setting (all South Africa) the summary risk ratio was 3.16 (95% CI 2.31, 4.32), and statistical heterogeneity 35%. Sub-group analysis by study design (case control vs. cohort) did not explain residual heterogeneity within tuberculosis burden country strata, nor overall. The difference in relative risk between the two tuberculosis burden country groups was not statistically significant (*p* = 0.10) after omitting the outlier study (Fig. 3).

**Fig. 4:**
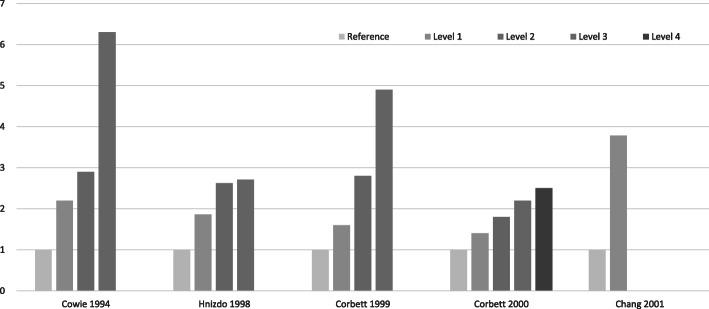
Tuberculosis risk/odds ratio (y-axis) by severity of silicosis. Definition of severity “level” varies by study. See Table 3 for detail

With regard to exposure-response, Table 3 summarises the findings of five studies which used grade of silicosis severity as the exposure, four cohort studies and one case control study. Since the ordinal scale did not allow pooling, a graphic synthesis is provided in Fig. 4. Two studies included some minor scale grades of the ILO classification for silicosis (0/1, 1/0, 1/1 and >  1/1), i.e. distinguished borderline categories from more advanced disease [50, 51], while a third used only the major scale grades (0, 1, 2 and 3) [48]. Hnizdo et al. [49] based their exposure-response analysis on subradiological (histological) silicosis and active tuberculosis detected at autopsy. Of note is that just under half to two thirds of the identified silicosis cases in these studies were at the lowest grades, i.e. ILO 1/0 or 1/1, or “negligible” to “slight” on histology. All controlled for age. Four were South African studies of goldminers - two controlled for HIV directly [50, 51], one covered a low HIV period [48] and the other a low HIV prevalence population [49]. Chang et al. [52] used progressive massive fibrosis as an indirect measure of severity. Table 3 and Fig. 4 shows a consistent monotonic increase in the risk or odds of tuberculosis with increasing grade of silicosis.

**Table 3:** Risk or odds of tuberculosis by severity of silicosis

Study by date of publication (effect measure)	Cowie 1994 (IR) [48]	Hnizdo 1998 (RR) [49] (*Autopsy grading*)	Corbett 1999 (OR) [50]	Corbett 2000 (RR) [51]	Chang 2001 (RR) [52]
Adjusted/controlled for. N	Age, date of CXR. *N* = 1153	Age, smoking. *N* = 2255	Age, HIV, duration employed, dusty job. *N* = 561	Age, HIV, duration employed, underground/surface job. *N* = 4022	Age, sex, smoking. *N* = 707
Grades or markers of extent of silicosis	None (*n* = 335): 1.0 (0, 2.0) ILO 1 (*n* = 418): 2.2 (0.7, 3.6) ILO 2 (*n* = 355): 2.9 (1.1, 4.6) ILO 3 (*n* = 45): 6.3 (0, 13.4)	None (*n* = 577): 1.00 Negligible (*n* = 310): 1.86 (0.97, 3.58) Slight (*n* = 196): 2.62 (1.36, 5.03) Moderate/ marked(*n* = 213): 2.71 (1.41, 5.20)	None (*n* = 340): 1.00 ILO 0/1 (*n* = 69): 1.6 (0.86, 2.90) ILO 1/0 (*n* = 48): 2.8 (1.24, 6.46) ILO > 1/1 (*n* = 90): 4.9 (2.32, 10.58)	None (*n* = 2924): 1.00 ILO 0/1 (*n* = 460): 1.4 (1.0, 2.2) ILO 1/0 (*n* = 212): 1.8 (1.0, 3.0) ILO 1/1 (*n* = 156): 2.2 (1.3, 3.7) ILO > 1/1 (*n* = 197): 2.5 (1.6, 4.0)	PMF (*n* = 141) vs no PMF (*n* = 566): 3.78 (2.24, 6.35)

95% confidence intervals in parentheses. *IR* incidence rate (annual), *RR* relative risk, rate ratio or risk ratio (see Table 1), *OR* odds ratio;

*CXR* chest x-ray, *ILO* International Labour Organization, *PMF* progressive massive fibrosis

Using the GRADE schema (Table 4) we rated our confidence in the evidence for a strong aetiological association (relative risk > 2.5) between silicosis and tuberculosis as high. This judgement was based on a low risk of bias (following close consideration of confounding and a sensitivity analysis), a consistent, large effect size, directness and the presence of an observed exposure response gradient. To reduce across-study bias, all study outcomes were reported. However, there were too few studies to perform a funnel plot to exclude publication bias [57].

**Table 4:** Silicosis and tuberculosis (adjusted and crude GIV), GradePro schema

Certainty assessment							No. of participants	Effect	Certainty
Country TB burden. No. of studies	Study design	Risk of bias ^a^	Inconsistency ^b^	Indirectness ^c^	Imprecision ^d^	Other considerations ^e^		Pooled relative risk (95% CI)	
All 8	Observational	Not serious	Not serious	Not serious	Not serious	Strong association, dose response gradient	66,982	3.65 (2.79, 4.78)	⨁⨁⨁⨁ HIGH
Low/intermediate 4	Observational	Not serious	Not serious	Not serious	Not serious	Strong association, dose response gradient	59,091	4.68 (3.22, 6.80)	⨁⨁⨁⨁HIGH
High 4	Observational	Not serious	Not serious	Not serious	No serious	Strong association, dose response gradient	7891	3.16 (2.31, 4.32)	⨁⨁⨁⨁ HIGH

*GIV* generic inverse variance, *CI* confidence interval

^a^ Risk of bias: Not downgraded. We used the Newcastle-Ottawa Scale for cohort and case-control studies. We explored confounding in depth and determined that although confounding was approached differently across studies, the overall risk of bias from the studies did not warrant marking down overall

^b^ Inconsistency: Not downgraded. Overall: I^2^ reduced from 53% to a moderate 35% following removal of outlier [46]. Low/intermediate TB burden countries: I^2^ reduced from 49 to 14% following omission of the outlier. High TB burden countries: I^2^ a moderate 35%, not explained by study design

^c^ Indirectness: Not downgraded. Exact diseases of interest were studied, and the studies covered different industries and country populations

^d^ Imprecision: Not downgraded. Large sample sizes with 95% confidence intervals in the strong effect range

^e^ Other considerations: Large effect and dose response gradient. We marked up for large effect as the associations were strong (relative risk > 2.5 to very strong > 4), although with a wide range. While we cannot exclude the possibility that further studies in different settings might alter the overall effect estimate, the clear dose response in five studies suggests that this is unlikely

### Studies of the association between silica exposure and tuberculosis, controlling for silicosis

**Fig. 5:**
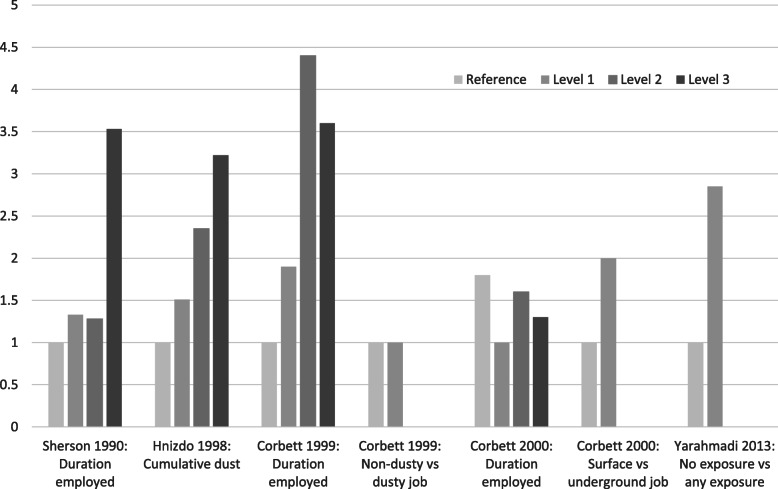
Tuberculosis risk/odds ratio (y-axis) by dust exposure, occupation or duration employed, controlling for silicosis. Metric and definition of “level” vary by study. See Table 5 for detail

**Fig. 6:**
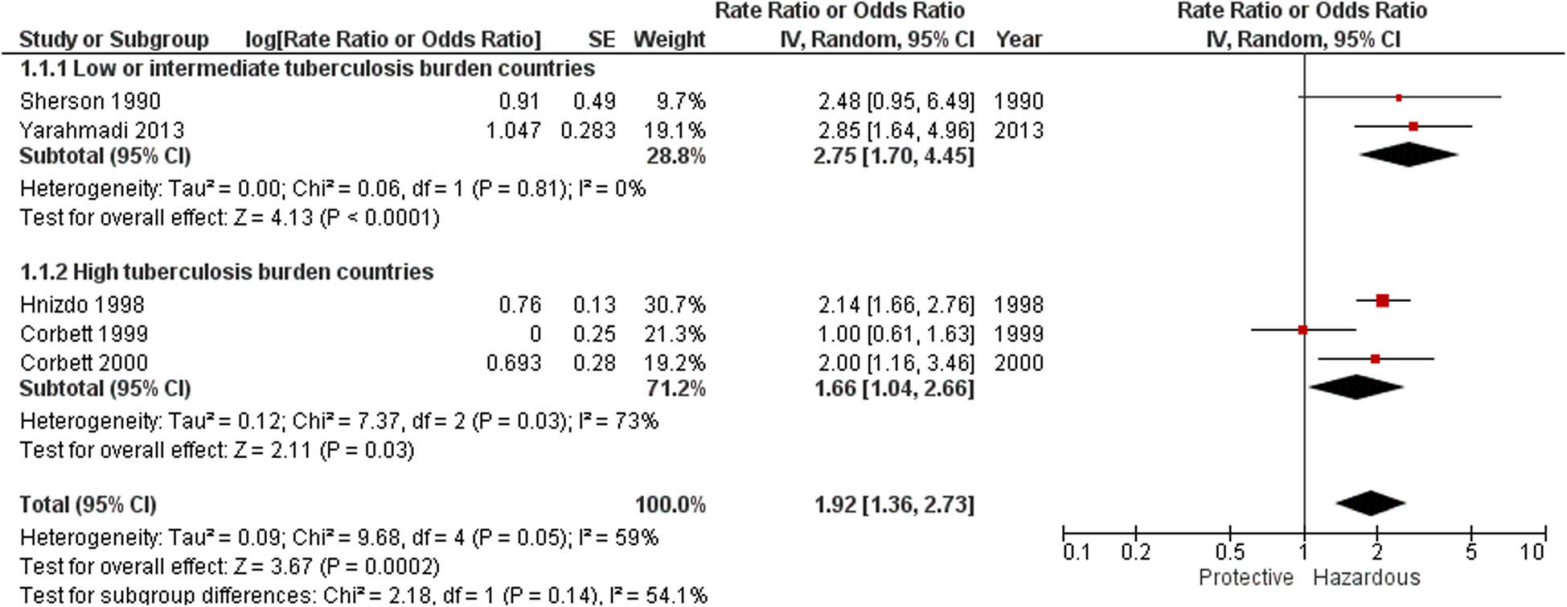
Forest plot: Studies of the association between silica exposure and tuberculosis, controlling for silicosis

Table 5 and Fig. 5 summarise the results of five studies which reported on silica exposure and controlled for silicosis, either by exclusion [46, 47], modelling [50, 51], or both [49]. The meta-analysis is presented in Fig. 6. In all of these studies, the association of silica exposure with tuberculosis persisted after excluding radiological silicosis (including at least one of the study analyses in those studies in which two metrics were used [50, 51]. The effect measures ranged from 1.00 (dusty occupation vs none) [50] to 2.85 (any vs no silica exposure) [54].

**Table 5:** Tuberculosis risk/odds by silica exposure, dustiness of occupation or exposure duration, controlling for silicosis

Sherson 1990 [47]	Hnizdo 1998 [49]	Corbett 1999 [50]	Corbett 2000 [51]	Yarahmadi 2013 [54]
SIR or RR ^a^ (95% CI)	RR (95% CI)	OR 95%CI)	RR (95%CI)	OR (95% CI)
Silicotics excluded.	White miners, > 10 years’ exposure; adjusted for silicosis ^b^, age, smoking.	Adjusted for silicosis, age, HIV.	Adjusted for silicosis, age, HIV.	Silicotics excluded. No other adjustment.
**Binary or summary effect measures**				
Duration of employment (yr), (RR) < 15: 1.00 > 15: 2.39 (0.96, 6.44)	Per 8 mg-yr/m^3 c^ 1.95 (1.58, 2.2)	Occupation (dusty job at diagnosis) No: 1.00 Yes 1.00 (0.62, 1.63)	Occupation (underground vs surface) No: 1.00 Yes: 2.00 (1.11, 3.33)	Silica exposure None: 1.00 Any: 2.85 (1.13, 3.42)
**Dose-response**				
Duration of employment (yr), SIR General male population: 100 0.15–14.5: 133 (57, 262) 15.0–24.5: 128 (26, 375) > 25: 353 (130, 768)	Cumulative dust (mg-yr/m^3^) quartile 1: 1.00 2: 1.51 (0.78, 2.91) 3: 2.35 (1.28, 4.32) 4: 3.22 (1.75, 5.90)	Duration of employment (yr) < 10: 1.00 10–14: 1.9 (1.07, 3.36) 15–19: 4.4 (2.45, 7.75) > 20: 3.6 (1.84, 7.12)	Duration of exposure (yr) 0–4: 1.8 (0.9, 3.5) 5–9: 1.00 10–19: 1.6 (1.0, 2.5) > 20: 1.3 (0.8, 2.3)	
Duration of employment (yr), (RR) 0.05–14.5: 1.00 15–24.5: 1.44 (0.38, 5.43) > 25: 3.9 (1.36, 11.23)^a^				

*RR* relative risk or rate ratio (see Table 1), *SIR* standardised incidence ratio, *CI* confidence interval, *RR* odds ratio

^a^ RR estimated for this review comparing silicotics with non-silicotics in the same cohort

^b^ Adjustment for radiological silicosis. Analysis by adjustment for *radiological or autopsy silicosis* yielded similar findings

^c^ RR for 1 mg-yr/m^3^ = 1.10 (1.06, 1.13). RR scaled to interquartile range (see Table 1 of the publication) of 10–17.99 mg-yr/m^3^ (as used in [58])

The summary relative risk, preferentially combining adjusted estimates where these were reported with crude estimates, was 1.92 (95% CI 1.36, 2.73), with I^2^ = 59%. This moderate statistical heterogeneity could be explained by study design difference: specifically, omission of the two case-control studies [50, 54] increased the relative risk to 2.13 (95% CI 1.70, 2.67) while reducing I^2^ to zero.

The summary relative risk for studies in low and intermediate burden tuberculosis countries was 2.75 (95% CI 1.70, 4.45) with I^2^ = zero. In high tuberculosis burden countries (South Africa) the summary risk ratio was 1.66 (95% CI 1.04, 2.66), and statistical heterogeneity high at I^2^ = 73%. As above, omission of the case-control study [50] increased the relative risk to 2.11 (95% CI 1.68, 2.66) while reducing I^2^ to zero. The difference in summary relative risk between the two tuberculosis burden country groups was not statistically significant (*p* = 0.14) (Fig. 6).

Using GRADE, we rated our confidence in the effect estimate for silica exposure and tuberculosis in the absence of silicosis as low (Table 6). Although exposure-response gradients and relative risks greater than two were observed in most of the analyses, this was not the case in all - we therefore did not mark up for magnitude of association nor exposure-response gradient. We considered there to be sufficient precision, consistency and directness not to mark down on these criteria. A risk of bias stemmed from the use of proxy metrics for silica exposure, with the potential for exposure misclassification bias [58]. Given that all of the studies except one were based on registers and not self-report, we regarded this risk as being non-differential with respect to tuberculosis, and therefore highly unlikely to have produced spurious associations. We therefore did not mark down further for risk of bias.

**Table 6:** Silica exposure controlled for silicosis and tuberculosis (adjusted and crude GIV), GradePro schema

Certainty assessment							No. of participants	Effect	Certainty
Country TB burden No. of studies	Study design	Risk of bias ^a^	Inconsistency ^b^	Indirectness ^c^	Imprecision ^d^	Other considerations		Pooled relative risk (95% CI)	
All 5	Observational	Not serious	Not serious	Not serious	Not serious	None	13,617	1.92 (1.36, 2.73)	⨁⨁◯◯LOW
Low/intermediate 2	Observational	Not serious	not serious	Not serious	Not serious	None	6879	2.75 (1.70, 4.45)	⨁⨁◯◯LOW
High 3	Observational	Not serious	not serious	Not serious	Not serious	None	6738	1.66 (1.04, 2.66)	⨁⨁◯◯LOW

*GIV* generic inverse variance, *CI* confidence interval

^a^ Risk of bias: Not downgraded. The overall risk of bias was low across four out of five studies measuring silica exposure. Non-differential exposure misclassification arising from use of proxies for silica exposure is likely to reduce rather than increase any true effect

^b^ Inconsistency: Not downgraded. Overall I^2^ reduced from 59% to zero after adjustment for study design by omission of the two case-control studies (one of which had a high risk of bias). Low/ intermediate TB countries): I^2^ = 0%. High TB countries: I^2^ was reduced from 73% to zero after adjusting for study design by omission of the one case-control study

^c^ Indirectness: Not downgraded as exposure metrics were well established proxies of silica exposure, the exact disease of interest was studied, and the studies covered miners in South Africa, foundry workers in Denmark and a variety of silica exposure occupations in Iran

^d^ Imprecision: Not downgraded. Large sample sizes and 95% confidence intervals exclude the null, i.e. sufficient precision

### Silicosis grade threshold for increased risk of tuberculosis

For radiological silicosis, an increase in the risk or odds of tuberculosis was seen at the ILO profusion grade 1/0 in two studies relative to the stratum of no silicosis [50, 51] (Table 3, Fig. 4). A third study used only major ILO grades and found an increased relative risk of 2.2 (95% CI 0.7, 3.6) at ILO grade 1 and 2.9 (95% CI 1.1, 4.6) at grade 2.

Threshold effects were difficult to infer from the four studies of silica exposure that controlled for silicosis and provided exposure-response gradients (Table 5) [47, 49–51]). One showed no duration effect [51], while in the two that did, the first increment in risk was estimated across wide strata: 10–14 vs <  10 years [50] and > 15 years vs 0.5–14.5 years [47]. The same applied to the one study which measured cumulative dust exposure, i.e. 10–14 mg-years/m^3^ vs < 9 mg-years/m^3^ [49].

## Discussion

### Summary of evidence

We have high confidence on the GRADE schema that further evidence would not change the conclusion that silicosis strongly increases the risk of tuberculosis, i.e. with a relative risk > 2.5 With regard to silica exposure controlling for radiological silicosis, the evidence suggests an elevated risk of tuberculosis with a exposure-response gradient. However, our confidence in the effect estimate is low and future studies, particularly those with more accurate measures of silica exposure, may change this estimate.

### Overall completeness and applicability of evidence

This is to our knowledge the first review to provide a meta-analysis and critically summarise the evidence of the association between silica exposure, and separately radiological silicosis, and pulmonary tuberculosis. The evidence covered spans the 1960s to 2013, with a large proportion of silicosis cases in the lower ILO (or histological severity) grades, distinguishing it from that of the first part of the century characterised by very high levels of silica dust exposure, severe silicosis and untreatable tuberculosis.

The co-modelling of silicosis and silica exposure in one study (which used both radiological and autopsy findings) [49] allowed an estimate of the combined independent effects of silicosis and cumulative dust exposure. The risk of tuberculosis in silicotic miners in the highest category of dust exposure was 13.4 times greater (multiplying the independent effects of 4.18 and 3.22 for silicosis and dust exposure category, respectively), than that of miners without silicosis in the lowest category of dust exposure.

Exposure-response gradients showed an increased risk of tuberculosis at an early radiologic grade of silicosis [50, 51]. Further, since a significant proportion of silicosis is subradiological [31, 32] the finding of an increased risk of tuberculosis at early histological grades [49] suggests an even lower threshold than that revealed radiologically. This is consistent with an earlier autopsy study showing an elevated proportion of tuberculosis even with a “slight degree of silicosis not detected radiologically in life” [59]. The excess risk of tuberculosis in silica exposed workers without radiological silicosis may thus be due to subradiological silicosis or cumulative silica dust each on its own, or to the combination. The implication of these findings is that radiological silicosis should not be required for attribution of the excess risk of tuberculosis to silica exposure in members of silica exposed workforces. However, there remains uncertainty about the threshold, in exposure or duration (as a proxy for exposure), at which excess risk of tuberculosis begins.

The review covered six countries, both high and low/intermediate tuberculosis burden, using different metrics of exposure. The overall estimates of effect for both silicosis and silica controlling for silicosis was higher in low/intermediate tuberculosis countries, arguably because of a relatively low baseline or population incidence of tuberculosis. However, only one high tuberculosis burden country, South Africa, was included. Workers on the South African gold mines and particularly black miners have a history of high rates of tuberculosis related to the migrant labour system and deep level gold mining going back 130 years. In addition to silica exposure, this system has entailed oscillation between mines and rural areas and congregate exposures in transport, accommodation and underground work, greatly aggravated by the HIV epidemic from the 1990s onwards [60, 61]. Studies are needed from other high tuberculosis burden countries such as India, China, Indonesia and Brazil, to determine if their patterns of risk differ.

A range of industries, occupations and settings were covered – gold mining, iron and steel foundries and caisson construction work [55] with one population based study covering a wide range of occupations [54]. This is, however, still relatively low coverage of the full range of occupations involving significant silica exposure, such as construction, agriculture, ceramics, stone work, sandblasting and fabrication of artificial stone. Besides differences in local tuberculosis epidemiology, differences in industry-associated potency factors for silicosis [62] may be relevant to the silica-tuberculosis association.

There has been recent international awakening to the fact that control of silica dust is important not only for preventing silicosis but also for control of tuberculosis [17, 18]. The summary relative risk (excluding one outlier study) of 3.65 (lower confidence limit 2.79) for silicosis and tuberculosis is of the same order as that of other common risk factors for tuberculosis [42] with the exception of HIV which is associated with a very high risk [39]. However, the impact of silica exposure on miners goes beyond the workforce. A modelling study of South African gold mining communities concluded that miners and members of mining communities contributed a disproportionately large share of new tuberculosis cases nationally in relation to their proportion of the population, although the main transmission impact was local [63].

### Potential biases in the review process

Potential biases in the review process were controlled via a structured and transparent approach. The study protocol followed PRISMA and was registered on Prospero. Structured searches of PUBMED and EMBASE were undertaken and articles selected independently by two subject experts and agreed by consensus. Studies were limited to those published after 1970 in English; however, only one potentially relevant abstract was identified among those records not reported in English. A structured tool for assessment of risk of bias was applied independently by two subject experts and agreed by consensus. In-depth exploration of confounding was undertaken along the lines recently recommended [64]. In studies with more than one outcome, all were considered; however, there were too few studies to explore for publication bias. Finally, GradePro was used to assess the degree of confidence in the effect estimate for the two primary associations studied.

### Agreements and disagreements with other studies or reviews

The findings are consistent with those from mortality studies. Workers in silica exposed occupations have been found to have standardised mortality ratios (SMRs) for tuberculosis ranging from 329 (95% CI 233, 452) [65] to 2175 (95% CI 1837, 2556) [66]. Studies specifically of radiological silicosis have shown similarly elevated SMRs, reaching 564 (95% CI 411, 754) in silicotic subjects who had claimed workers’ compensation [67].

We excluded mortality studies from the review and meta-analysis for several reasons. Death certificate studies generally provide no information on how the initial tuberculosis diagnosis was made. Substantial misclassification of tuberculosis as the cause of death has been shown in the South African gold mining context of very high rates of silicosis, tuberculosis and HIV infection [68]. Further, silicosis may contribute to tuberculosis mortality without an association with incident tuberculosis, for example, by creating diagnostic confusion and delaying TB treatment [69], or acting as an effect modifier by aggravating the course of tuberculosis through coexistent fibrosis, lung function impairment or reducing the effectiveness of standard treatment. Some such effect is suggested by the finding of the case fatality rate among those being treated for tuberculosis to be three times higher in silicotics than non-silicotics [70].

The findings are also consistent with cross-sectional studies (Additional File 1, Table S3) which reported positive associations between tuberculosis and silicosis and metrics of silica exposure controlling for silicosis [38, 58]. However, we excluded cross-sectional studies because of potential biases arising from exposure or disease related selection of workers with silicosis or tuberculosis out of the workforce prior to the sampling; underestimation of effect by including only prevalent active tuberculosis; or uncertainty associated with a diagnosis of past tuberculosis based on self-report or old radiologic features and the temporal relationship of such diagnosis with silica exposure.

The increased risk of pulmonary tuberculosis associated with silica exposure found in observational epidemiologic studies is supported by animal experiment studies. From as early as the first decades of the twentieth century, experiments on small laboratory animals showed that exposure to silica dust and tubercle bacilli resulted in greater proliferation of bacilli, faster development of tuberculosis or more severe disease in exposed compared to control animals [71–75]. More recent work has shown that silica exposure markedly increases susceptibility to mycobacterial infection in mice, and that macrophages transplanted from exposed into unexposed mice reproduce this susceptibility without concomitant dust exposure [76]. Biological plausibility has been further strengthened by the demonstration or elaboration of possible underlying biologic mechanisms [77, 78]. Silica inhalation or silicosis should therefore be considered an effect modifier of the progression of tuberculosis from recent infection or latent infection to active disease.

### Considerations in future research

Interpretation of studies of silica exposure, silicosis and tuberculosis needs to take the phenomenon of subradiological silicosis into account in terminology, causal contrast and interpretation. Computerised tomographic (CT) scanning may improve sensitivity to a variable degree [31] but remains impractical for large scale studies and medical surveillance.

There is a need for studies which are able to provide a more accurate measure of the dose of respirable silica dust, controlling for radiological silicosis, and its relationship to incident pulmonary tuberculosis. Given that exposure is a continuous variable, the goal should be a exposure-response curve, as has been achieved for respirable silica and silicosis [79]. This is best achieved through cohort studies, ideally prospective. Such studies would provide a more accurate estimate of the size of the effect of a given cumulative silica exposure on tuberculosis risk than presently available. For prevention purposes, a central question is the exposure threshold below which there is no excess risk of tuberculosis. The question of whether elimination of radiological silicosis, i.e. under current protective dust standards, would be sufficient to protect against tuberculosis, is a corollary of this.

Understanding of time dependent phenomena is also required. This includes the effect of short-term high intensity silica exposure on tuberculosis risk as opposed to long-term lower exposure; or the extent to which, in the absence of radiological silicosis, excess risk persists once silica exposure has ended.

More explicit attention to potential confounding is needed, in this case ensuring that risk factors for tuberculosis are evenly distributed across comparison groups or controlled in the analysis. Even if age is adjusted for, general populations may be an inadequate control for silica-exposed populations or silicosis cases without additional information on confounders. Including relevant covariates would also allow assessment of effect modification of the silica exposure/silicosis tuberculosis thresholds by covariates such as age, HIV, smoking and diabetes.

## Conclusions

The study provides to our knowledge the first systematic review of the epidemiological evidence for an association identified at least a century ago, viz. that occupational inhalation of silica dust increases the risk of pulmonary tuberculosis in co-exposed populations. In the current era, even with less severe forms of silicosis in traditional dusty industries, the evidence remains strong for a substantially elevated risk of tuberculosis in those with radiologically diagnosed disease. While there is evidence for an elevated risk of tuberculosis in those who have not been radiologically diagnosed with silicosis, the effect size is subject to uncertainty. Further studies are needed to characterise this effect, and particularly the exposure threshold that would avoid an excess risk of tuberculosis.

## Supplementary Information

**Additional file 1: Table S1.** Search strings. **Table S2.** Newcastle-Ottawa quality assessment scales. **Table S3.** Exclusions on full article. **Supplementary** **Note**: Consideration of confounding and bias. **Table S4.** Potential confounders of the association between silica, silicosis and tuberculosis. **Table S5.** E-values for potential confounders of the association between silicosis and tuberculosis.

## Data Availability

The data that support the findings of this study are all derived from published material as referenced in the main article or Supplementary Material.

## Abbreviations

CI: Confidence interval
ILO: International Labour Organization
HIV: Human immunodeficiency virus
NOS: Newcastle Ottawa Scale
PRISMA: Preferred Reporting Items for Systematic Reviews and Meta-Analyses
SMRs: Standardised mortality ratios
WHO: World Health Organization
