# Supplementary material for: The association between silica exposure, silicosis and tuberculosis: a systematic review and meta-analysis
Source: BMC Public Health. 2021 May 20;21:953. doi: 10.1186/s12889-021-10711-1 (PMC8136154; doi:10.1186/s12889-021-10711-1)
Supplement: Supplementary file 1 — Additional file 1: Table S1. Search strings. Table S2. Newcastle-Ottawa quality assessment scales. Table S3. Exclusions on full article. Supplementary Note: Consideration of confounding and bias. Table S4. Potential confounders of the association between silica, silicosis and tuberculosis. Table S5. E-values for potential confounders of the association between silicosis and tuberculosis. [file 12889_2021_10711_MOESM1_ESM.docx]

**ADDITIONAL FILE 1**

**Table S1 Search Strings**

**PUBMED (1970 – 2020)**

| **Search** |  | **Query** | **Items found** |
| --- | --- | --- | --- |
| [#6](http://www.ncbi.nlm.nih.gov/pubmed) |  | Search (((#1 AND #4) NOT (animals [mh] NOT humans [mh]))) AND ("1970/01/01"[Date - Publication] : "2020/04/20"[Date - Publication]) | [1454](http://www.ncbi.nlm.nih.gov/pubmed/?cmd=HistorySearch&querykey=6) |
| [#5](http://www.ncbi.nlm.nih.gov/pubmed) |  | Search (#1 AND #4) | [2372](http://www.ncbi.nlm.nih.gov/pubmed/?cmd=HistorySearch&querykey=5) |
| [#4](http://www.ncbi.nlm.nih.gov/pubmed) |  | Search (#2 OR #3) | [105591](http://www.ncbi.nlm.nih.gov/pubmed/?cmd=HistorySearch&querykey=4) |
| [#3](http://www.ncbi.nlm.nih.gov/pubmed) |  | Search (silicon dioxide[mh] OR silicon dioxide[tiab] OR silica[tiab] OR tridymite[tiab] OR cristobalite[tiab] OR quartz[tiab]) | [90140](http://www.ncbi.nlm.nih.gov/pubmed/?cmd=HistorySearch&querykey=3) |
| [#2](http://www.ncbi.nlm.nih.gov/pubmed) |  | Search (silicosis[mh] OR silicosis[tiab] OR silicoses[tiab] OR pneumoconiosis[mh] OR pneumoconiosis[tiab] OR pneumoconioses[tiab]) | [19715](http://www.ncbi.nlm.nih.gov/pubmed/?cmd=HistorySearch&querykey=2) |
| [#1](http://www.ncbi.nlm.nih.gov/pubmed) |  | Search (tuberculosis[mh] OR tuberculosis[tiab] OR tb[tiab]) | [213319](http://www.ncbi.nlm.nih.gov/pubmed/?cmd=HistorySearch&querykey=1) |

**EMBASE (1970 – 2020)**

| **No.** | **Query** | **Results** |
| --- | --- | --- |
| #6 | #1 AND #4 AND [1970-2020]/py | 1242 |
| #5 | #1 AND #4 | 1478 |
| #4 | #2 OR #3 | 87691 |
| #3 | 'silicon dioxide'/de OR 'silicon dioxide':ab,ti OR silica:ab,ti OR cristobalit*:ab,ti OR quartz:ab,ti OR tridymite:ab,ti OR tridimite:ab,ti OR 'silicone dioxide':ab,ti OR 'hydrated silicate':ab,ti OR reolosil:ab,ti OR smectite:ab,ti | 75420 |
| #2 | 'silicosis'/de OR silicosis:ab,ti OR pneumosilicosis:ab,ti OR pneumonosilicosis:ab,ti OR 'pneumoconiosis'/de OR pneumoconiosis:ab,ti | 15044 |
| #1 | 'tuberculosis'/de OR tuberculosis:ab,ti OR tb:ab,ti | 209108 |

**Table S2 Newcastle-Ottawa quality assessment scales**

**Case control studies**

Note: A study can be awarded a maximum of one star for each numbered item within the Selection and Exposure categories. A maximum of two stars can be given for Comparability.

**Selection**

1) **Is the case definition adequate?**

a) yes, with independent validation **¯**

b) yes, e.g. record linkage or based on self reports

c) no description

2) **Representativeness of the cases**

a) consecutive or obviously representative series of cases **¯**

b) potential for selection biases or not stated

3) **Selection of Controls**

a) community controls **¯**

b) hospital controls

c) no description

4) **Definition of Controls**

a) no history of disease (endpoint) **¯**

b) no description of source

**Comparability**

1) Comparability of cases and controls on the basis of the design or analysis

a) study controls for _HIV______________ (Select the most important factor.) **¯**

b) study controls for any additional factor AGE **¯** (This criteria could be modified to indicate specific

control for a second important factor.)

**Exposure**

1) **Ascertainment of exposure**

a) secure record (e.g. surgical records) **¯**

b) structured interview where blind to case/control status **¯**

c) interview not blinded to case/control status

d) written self report or medical record only

e) no description

2) **Same method of ascertainment for cases and controls**

a) yes **¯**

b) no

3) **Non-Response rate**

a) same rate for both groups **¯**

b) non respondents described

c) rate different and no designation

**Cohort studies**

Note: A study can be awarded a maximum of one star for each numbered item within the Selection and Outcome categories. A maximum of two stars can be given for Comparability

**Selection**

1) **Representativeness of the exposed cohort**

a) truly representative of the average _______________ (describe) in the community **¯**

b) somewhat representative of the average ______________ in the community **¯**

c) selected group of users eg nurses, volunteers

d) no description of the derivation of the cohort

2) **Selection of the non exposed cohort**

a) drawn from the same community as the exposed cohort **¯**

b) drawn from a different source

c) no description of the derivation of the non exposed cohort

3) **Ascertainment of exposure**

a) secure record (eg surgical records) **¯**

b) structured interview **¯**

c) written self report

d) no description

4) **Demonstration that outcome of interest was not present at start of study**

a) yes **¯**

b) no

**Comparability**

1) **Comparability of cohorts on the basis of the design or analysis**

a) study controls for _____________ (select the most important factor) **¯**

b) study controls for any additional factor **¯** (This criteria could be modified to indicate specific

control for a second important factor.)

**Outcome**

1) **Assessment of outcome**

a) independent blind assessment **¯**

b) record linkage **¯**

c) self report

d) no description

2) **Was follow-up long enough for outcomes to occur**

a) yes (select an adequate follow up period for outcome of interest) **¯**

b) no

3) **Adequacy of follow up of cohorts**

a) complete follow up - all subjects accounted for **¯**

b) subjects lost to follow up unlikely to introduce bias - small number lost - > __20__ % (select an

adequate %) follow up, or description provided of those lost) **¯**

c) follow up rate < _20___% (select an adequate %) and no description of those lost

d) no statement

**Table S3 Exclusions on full article**

|  | **First author, year, (country)** | **Reason for exclusion** | **Relevant finding** |
| --- | --- | --- | --- |
| 1 | Paul 1961 [Northern Rhodesia (Zambia)] | Cohort study but TB diagnosis unspecified, most likely CXR only. | Silicosis-TB risk ratio declined from 41 in 1950 to 30 in 1959. |
| 2 | Gzrybowski 1971  (Canada) | Case register. “Pneumoconiosis” unspecified other than that “most … have silicosis”. No other information. | > 20-fold annual TB incidence rate relative to general population. |
| 3 | Snider 1978 | Editorial | N/A |
| 4 | Morgan 1979 | Editorial response | N/A |
| 5 | Rosenman 1996  (USA) | Case-control study but only occupations/industries with elevated odds of TB reported. | Silica using industries with elevated ORs for TB: quarrying, pottery and related products, non-metallic mineral and stone products; ship, boat building and repair. |
| 6 | Kleinschmidt 1997 (South Africa) | No identifiable cohort. Cross-sectional sample with retrospective calculation of TB incidence rate from register. | Range of adjusted TB IRRs for dusty vs low dust occupations: 1.3 to 2.56; IRR for silicosis 1.54 (95% CI 1.00, 2.37). No association of TB with duration of employment. |
| 7 | Corbett 2003 (South Africa) | Overlap with Corbett et al. 2000. | As for Corbett et al. 2000. |
| 8 | Tiwari 2007 (India) | Cross-sectional study. TB on CXR and clinical history. | Prevalence of TB higher with longer duration of silica exposure. |
| 9 | Leung 2007 (Hong Kong). | No non-silicosis comparison group. | N/A |
| 10 | teWaterNaude 2006 (South Africa) | Cross-sectional study. TB on CXR (active or old) and/or self-reported history. | Controlling for silicosis, POR for TB per interquartile range of cumulative respirable quartz exposure: 1.47 (95% CI 1.14, 1.89). |
| 11 | Xia 2014 (China) | Silicosis and silicotuberculosis register 2008-2013. | N/A |
| 12 | Hui 2014 (China) | Overlap with database analysed in Li et al. 2011. | Silicosis associated with incident TB but wide confidence interval based on 4 silicosis cases. |
| 13 | Farazi 2015 (India) | Outcome LTBI rather than TB. | N/A |
| 14 | Ngosa 2016 (Zambia) | Cross-sectional study. TB on CXR or positive sputum (register). Silicosis not  recorded. | Dose response relationship between cumulative silica dust exposure and TB. |

Note: TB, tuberculosis; CXR, chest x-ray; N/A, not applicable; OR, odds ratio; IRR, incidence rate ratio; CI, confidence interval; POR, prevalence odds ratio; LTBI, latent tuberculosis infection.**Supplementary Note: Consideration of confounding and bias**

Table S4 summarises the potential confounders in studies of silicosis and/or silica exposure and tuberculosis. All studies adjusted for age, the most important confounder because of its association with silicosis (Nelson et al. 2010; Knight et al. 2020), duration of silica exposure, and tuberculosis (Kleinschmidt and Churchyard 1997; Corbett et al. 1999; Donald et al. 2010)

Other potential confounders are known predictors of tuberculosis, notably HIV, congregate settings, socioeconomic status, undernutrition, indoor air pollution, smoking, heavy alcohol use and diabetes (Lonnroth et al.2020; Martinson et al. 2011; Narasimhan et al. 2013).

With regard to smoking, one narrative review reported “limited evidence” of an association between smoking and silicosis (Hessel et al. 2003). Pack-years of smoking, although not smoking intensity nor smoking calendar period, would to some degree be controlled by controlling for age as above. Two included studies controlled for smoking (Hnizdo and Murray 1998; Yarahmadi et al.1993) with persistence of the association between silicosis or silica exposure and tuberculosis.

HIV is a very strong risk factor for progression to active tuberculosis (Martinson et al. 2011), but is not known to be associated with silicosis. In the South African studies, HIV was controlled for directly in two of the studies (Corbett et al. 1999, 2000). A third study (Cowie 1994) was conducted during the period 1984-1991 when HIV prevalence was very low in South Africa. The fourth study (Hnzido and Murray 1998) covered only white miners (higher occupational levels) who have had very low HIV prevalences throughout the epidemic (Ndlovu et al. 2018).

Congregate settings (workplace, accomodation or transport), socioeconomic status, indoor air pollution and undernutrition are unlikely to be a confounders in studies within the same workforce or industry where individuals without silicosis or with shorter durations of service were used as controls (Westerholm et al.1986, Cowie 1994, Hnizdo and Murray 1998; Corbett et al. 1999, 2000). Confounding on these variables is, however, a possibility in analyses based on general population samples or controls (Sherson and Lander 1990; Chang et al. 2001; Li et al. 2011, Yarahmadi et al. 2013). Of the latter studies, one adjusted for income (Li et al. 2011) and another for education (Yarahmadi et al. 2013) with persistence of an effect. In the Sherson and Lander study (1990), use of foundry (industry) controls rather than the general population yielded a crude relative risk of 8.3, close to the standardised incidence rate of 10 calculated using a general population control (Table 2), suggesting little additional confounding through use of the latter.

There is no information on the association between diabetes or alcohol abuse and silicosis or silica exposure. In the one study that adjusted for diabetes (Li et al. 2011) a strong association of silicosis with tuberculosis persisted.

A further consideration is that since dose-response gradients were shown in almost all of silicosis and silica exposure studies, the confounder would itself need to be independently associated with increasing grades of silicosis or duration or intensity of silica exposure. After controlling for age, we consider it highly implausible that the factors considered would meet this requirement.

The above considerations do not exclude residual or unmeasured confounding. We therefore calculated E-values (VanderWeele and Ding 2017) (Table S5) to illustrate the size of the association with both silicosis and tuberculosis that potential confounders would need to reach to efface the associations found. The E-values for confounding that would explain the effect estimates found all implausibly large. However, confounding that resulted in a lower confidence limit below the null would be plausible in Yarahmadi et al. 2013.

Besides confounding, ascertainment bias may occur if workforces exposed to silica dust or individuals with silicosis are subject to more regular screening for tuberculosis. Such bias is unlikely where the whole workforce (irrespective of whether they have previously been diagnosed with silicosis or not) is subject to periodic screening for lung disease, as was the case in the South African mining studies (Cowie 1994, Hnzido and Murray 1998; Corbett et al. 1999, 2000) or that of Swedish mining and foundry workers (Westerholm et al. 1986). Such bias may arise where a cohort with silicosis is compared to the general population, as in Chang et al. (2001) and Sherson and Lander (1990). Information bias may occur where individuals with tuberculosis are interviewed in a community study about past silica exposure where case status is known to the interviewer (Yarahmadi et al. 2013).

**Table S4** **Potential confounders of the association between silica, silicosis and tuberculosis**

| **Variable** | **Associ-ation with TB** | **Association with silicosis or silica exposure** | **Potential confounder?** | **Control in studies reviewed** |
| --- | --- | --- | --- | --- |
| Age | Yes | Yes | Yes | All |
| HIV | Yes | No known association with silicosis, but little published evidence. | Possible in South African studies from mid-1990s onwards. | Adjusted for in two studies (Corbett et al. 1999, 2000). One study covered pre-HIV period (Cowie 1994). Fourth study was of white (supervisory level) miners with low HIV prevalence. |
| Socio-economic status (SES); congregate settings; undernutrition; indoor air pollution. | Yes | Partly. Dusty manual work likely to be associated with lower SES, and congregate settings as in mining. | Possible where general population is used as low/no silicosis or dust exposure reference. | In five studies, comparison was non-silicotic or silica exposed workers in same occupation or industry (Westerholm et al. 1986; Cowie 1994; Hnizdo and Murray 1998; Corbett et al. 1999, 2000). In two general population studies, income (Li et al. 2011) and education (Yarahmadi et al. 2013) was controlled respectively. |
| Smoking | Yes | Limited evidence for positive association (Hessel et al. 2003) | Yes | Smoking adjusted for in Hnizdo and Murray 1998. Controlling for age would partially control for pack-years of smoking. |
| Heavy alcohol use | Yes | Sparse evidence of no association (Zhang et al. 2010) | Yes | Controlling for age would partially control for cumulative alcohol intake. |
| Diabetes | Yes | No evidence | Not known | Controlled for in one study (Li et al. 2011) |

**Table S5** **E-values for potential confounders of the association between silicosis and tuberculosis**

| **Study by date of publication and first author** | **Estimate (95% CI)** | **E-Value:**  **estimate (lower confidence limit)** |
| --- | --- | --- |
| Westerholm 1986 | OR 32.99 (4.50, 241.58)^a^ | 10.95 (3.66) |
| Sherson 1990 | SIR 1000 (272, 2561) | 1 948 (488) |
| Chang 2001 | SIR 4.9^b^ | 9.57 |
| Li 2011 | HR 5.82 (2.17, 15.6) | 11.11 (3.76) |
| Cowie 1994 | RR 2.8 (1.9, 4.1) | 5.04 (3.2) |
| Hnizdo 1998 | RR 3.96 (2.59, 6.06) | 7.38 (4.61) |
| Corbett 1999 | OR 4.90 (2.32, 10.58) | 9.27 (4.06) |
| Corbett 2000 | ILO = 1/1: RR 2.2 (1.3, 3.7)  ILO > 1/1: RR 2.5 (1.6, 4.0) | 3.82 (1.92)  4.43 (2.57) |
| Yarahmadi 2013 | RR 2.85 (1.13-3.42) | 5.14 (1.51) |

Note: CI, confidence interval; OR, odds ratio; SIR, standardised incidence ratio; HR, hazard ratio; RR, relative risk, rate ratio or risk ratio (see Table 1); ILO, International Labour Organisation.

E-value = RR + √ [RR x (RR- 1)] (VanderWeele and Ding 2017). Applied example (data from Li et al. 2011): The observed risk ratio of 5.82 could be explained away by an unmeasured confounder that was associated with both the exposure and the outcome by a risk ratio of 11.11 each, above and beyond the measured confounders, but weaker confounding could not do so; the confidence interval could be moved to include the null by an unmeasured confounder that was associated with both the exposure and the outcome by a risk ratio of 3.76 each, above and beyond the measured confounders, but weaker confounding could not do so. (For OR of common outcomes, RR is substituted by √OR.)

^a^ OR and CI estimated for this review.

^b^ No CI provided.

^c^ OR for silicosis > 1/1 not provided. ORs for ILO 1/1 and > 1/1 included here.

**References used in Additional File**

Chang KC, Leung CC, Tam CM. Tuberculosis risk factors in a silicotic cohort in Hong Kong. 2001. Int J Tuberc Lung Dis 5(2):177-184, PMID: 11258512.

Corbett EL, Churchyard GJ, Clayton T, Herselman P, Williams B, Hayes R, et al. 1999. Risk factors for pulmonary mycobacterial disease in South African gold miners: a case-control study. Am J Respir Crit Care Med 159(2):94-99, PMID: 9872824, https://doi.org/10.1164/ajrccm.159.1.9803048.

Corbett EL, Churchyard GJ, Clayton TC, Williams BG, Mulder D, Hayes RJ, et al. 2000. HIV infection and silicosis: the impact of two potent risk factors on the incidence of mycobacterial disease in South African miners. AIDS 14(17):2759-2768. PMID: 11125895, https://doi.org/10.1097/00002030-200012010-00016.

Corbett EL, Charalambous S, Fielding K, Clayton T, Hayes RJ, De Cock KM et al. 2003. Stable incidence rates of tuberculosis (TB) among human immunodeficiency virus (HIV)–negative South

African gold miners during a decade of epidemic HIV-associated TB. J Inf Dis 188(8):1156-1163, PMID: 14551886, https://doi.org/10.1086/378519.

Cowie RL. The epidemiology of tuberculosis in gold miners with silicosis. 1994. Am J Respir Crit Care Med 150(1):1460-1462, PMID: 7952577, https://doi.org/10.1164/ajrccm.150.5.7952577.

Donald PR, Marais BJ, Barry CE. 2010. Age and the epidemiology and pathogenesis of tuberculosis. Lancet 375(9729):1852-1854, PMID: 20488519, https://doi.org/10.1016/S0140-6736(10)60580-6.

Farazi A, Jabbariasl M. 2015. Silico-tuberculosis and associated risk factors in central province of Iran. Pan Afr Medical J 20:333, PMID: 26175823, https://doi.org/10.11604/pamj.2015.20.333.4993.

Grzybowski S, Fishaut H, Rowe J, Brown A. 1971. Tuberculosis among patients with various radiological abnormalities followed by the chest service. Am Rev Respir Dis 104(4):605-608, PMID: 5094062, https://doi.org.10.1164/arrd.1971.104.4.605.

Hessel PA, Gamble JF, Nicolich M. 2003. Relationship between silicosis and smoking. Scand J Work Environ Health 29(5):329-336, PMID: 14584513, https://doi.org/10.5271/sjweh.739.

Hnizdo E, Murray J. 1998. Risk of pulmonary tuberculosis relative to silicosis and exposure to silica dust in South African gold miners. Occup Environ Med 55(7):496-502. PMID: 9816385, https://doi.org/10.1136/oem.55.7.496.

Hu HY, Wu CY, Huang N, Chou YJ, Chang YC, Chu D. 2014. Increased risk of tuberculosis in patients with end-stage renal disease: a population-based cohort study in Taiwan, a country of high incidence of end-stage renal disease. Epidemiol Infect 142(1):191-199, PMID: 23510593, https://doi.org/10.1017/S0950268813000551.

Ioannidis JPA, Tan YJ, Blum MR. 2019. Limitations and misinterpretations of e-values for sensitivity analyses of observational studies. Ann Intern Med 170(2):108-111, PMID: 30597486,

https://doi-org/10.7326/M18-2159.

Kleinschmidt I, Churchyard G. 1997. Variation in incidences of tuberculosis in subgroups of South African gold miners. Occup Environ Med 54(9):636-641, PMID: 9423575, https://doi.org.0.1136/oem.54.9.636.

Knight D, Ehrlich R, Cois A, Fielding K, Grant A, Churchyard G. 2020. Predictors of silicosis in an industry wide study of working gold miners. BMC Public Health 20(1):829, PMID 32487111,

https://doi.org/10.1186/s12889-020-08876-2.

Leung CC, Yew WW, Law WS, Tam CM, Leung M, Chung YW, et al. 2007. Smoking and tuberculosis among silicotic patients. Eur Respir J 29(4):745-750, PMID:17182648,

https://doi.org/10.1183/09031936.00134706.

Li SY, Chen TJ, Chung KW, Tsai LW, Yang WC, Chen JY, et al. 2011. Mycobacterium tuberculosis infection of end-stage renal disease patients in Taiwan: a nationwide longitudinal study. Clin Microb Infec 17(11):1646-1652, PMID: 21375664, https://doi.org/10.1111/j.1469-0691.2011.03473.x

Lönnroth K, Castro KG, Chakaya JM, Chauhan LS, Floyd K, Glaziou P, et al. 2010. Tuberculosis control and elimination 2010-50: cure, care, and social development. Lancet 375(9728):1814-29, PMID: 20488524, https://doi.org/10.1016/S0140-6736(10)60483-7.

Martinson, NA. Hoffmann CJ, Chaisson RE. 2011. Epidemiology of tuberculosis and HIV: recent advances in understanding and responses. Proc Am Thorac Soc 8(3):288–293, PMID: 21653530,

https://doi.org/10.1513/pats.201010-440 064WR.

Morgan WK. 1979. The relationship between tuberculosis and silicosis. Am Rev Respir Med 1979; 119(2):319-320, PMID: 434603, https://doi.org.10.1164/arrd.1979.119.2.319.

Narasimhan P, Wood J, MacIntyre CR, Mathai D. 2013. Risk factors for tuberculosis. Pulm Med 2013:828939, PMID: 23476764, https://doi.org/10.1155/2013/828939.

Ndlovu N, Musenge E, Park SK, Girdler-Brown B, Richards G, Murray J. 2018. Four decades of pulmonary tuberculosis in deceased South African miners: trends and determinants. Occup Environ Med 75(11):767-775. PMID:29934377, <https://doi.org/10.1136/oemed-2017-104806>.

Nelson G, Girdler-Brown B, Ndlovu N, Murray J. 2010. Three decades of silicosis: disease trends at autopsy in South African gold miners. Environ Health Perspect 118(3):421-426, PMID:20194070,

https://doi.org/10.1289/ehp.0900918.

Ngosa K, Naidoo RN. 2016.The risk of pulmonary tuberculosis in underground copper miners in Zambia exposed to respirable silica: a cross-sectional study. BMC Public Health 2016;16(1):855, PMID: 27552992, https://doi.org/10.1186/s12889-016-3547-2.

Paul R. 1961. Silicosis in Northern Rhodesia copper mines. Arch Environ Health 2:96-109, PMID: 13733456, https://doi.org/10.1080/00039896.1961.10662823.

Rosenman KD, Hall N. 1996. Occupational risk factors for developing tuberculosis. Am J Ind Med 30(2):148-154, PMID: 8844044, https://doi.org/10.1002/(sici)1097-0274(199608)30:2%3C148::aid-ajim5%3E3.0.co;2-x.

Sherson D, Lander F. 1990. Morbidity of pulmonary tuberculosis among silicotic and nonsilicotic foundry workers in Denmark. J Occup Medicine 32(2):110-113, PMID: 2303918.

Snider D. 1978. The relationship between tuberculosis and silicosis. Am Rev Respir Med 118(3): 455-460, PMID: 707873, https://doi.org/10.1164/arrd.1978.118.3.455.

Tiwari RR, Sharma YK, Saiyed HN. 2007. Tuberculosis among workers exposed to free silica dust. Indian J Occup Environ Med 11(2):61-64. PMID: 21938217, https://doi.org/10.4103/0019-5278.34530.

VanderWeele TJ, Ding P. 2017. Sensitivity analysis in observational research: introducing the E-value. Ann Intern Med 167(4):268-274, PMID: 28693043, https://doi.org/10.7326/M16-2607.

Westerholm P, Ahlmark A, Maasing R, Segelberg I. 1986. Silicosis and risk of lung cancer or lung tuberculosis: a cohort study. Environ Res 41(1):339-350, PMID: 3757978, https://doi.org/10.1016/s0013-9351(86)80195-5.

Xia Y, Liu J, Shi T, Xiang H, Bi Y. 2014. Prevalence of pneumoconiosis in Hubei, China from 2008 to 2013. Int J Environ Res Public Health 11(9):8612-8621, PMID: 25158135, https://doi.org/10.3390/ijerph110908612.

Yarahmadi A, Zahmatkesh MM, Ghaffari M, Mohammadi S, Labbafinejad Y, Seyedmehdi SM, et al. 2013. Correlation between silica exposure and risk of tuberculosis in Lorestan Province of Iran. Tanaffos 12(2):34-40. PMID: 25191460.

Zhang M, Zheng YD, Du XY, Lu Y, Li WJ, Qi C, et al. 2010. Silicosis in automobile foundry workers: a 29-year cohort study. Biomed Environ Sci 23(2):121-129, PMID:20514987, htttps://doi.org/10.1016/S0895-3988(10)60041-4.
